# Supplementary material for: Research on supporting mechanism of ancillary service of PV system to grid energy efficiency based on multi-time and space-time operation
Source: PLoS One. 2022 May 13;17(5):e0268173. doi: 10.1371/journal.pone.0268173 (PMC9106158; doi:10.1371/journal.pone.0268173)
Supplement: S1 Table — (DOCX) [file pone.0268173.s001.docx]

| Abbreviation | Explanation of Nouns |
| --- | --- |
| SVG | Static Var Generator |
| P&O | Perturbation and Observation Method |
| MPPT | Maximum Power Point |
| MC | Monte Carlo method |
| RO | Robust Optimization |
| IES | Integrated Energy System |
| P2G | Power to Gas |
| EMS | Energy Management System |
| THD | Total Harmonic Distortion |
| LMS | Least Mean Square |
| NLMS | Normalized Least Mean Square |
| LLMS | Leaky Least Mean Square |
| CNN | Convolution Neural Networks |
| NSGA | Non-dominated Sorting Genetic Algorithm |
| CFSFDP | Clustering By Fast Search and Find Of Density Peaks |
| pu | Per unit |
| PVGU | Photovoltaic Generation Unit |
