# Supplementary material for: Research on supporting mechanism of ancillary service of PV system to grid energy efficiency based on multi-time and space-time operation
Source: PLoS One. 2022 May 13;17(5):e0268173. doi: 10.1371/journal.pone.0268173 (PMC9106158; doi:10.1371/journal.pone.0268173)
Supplement: Attachment — Submitted filename: Response to Reviewers.docx [file pone.0268173.s002.docx]

Dear Editor and Reviewers,

We are very grateful to you for giving us an opportunity to revise our manuscript. We appreciate you very much for your positive and constructive comments and suggestions on our manuscript entitled “Research on Supporting Mechanism of Ancillary Service of PV System to Grid Energy Efficiency Based on Multi-time and Space-time Operation”

We have studied reviewers’ comments carefully and tried our best to revise our manuscript according to the comments. The following are the responses and revisions I have made in response to the reviewers' questions and suggestions on an item-by-item basis. Thanks again for the hard work of the editor and reviewer!

Response to the comments of Reviewer #1

Comment: The authors should consider the harmonic issue and discusses how the proposed solution can mitigate the said problem. An extensive review of research in harmonic mitigation effort has been published in ["Advances in reduction of total harmonic distortion in solar photovoltaic systems: A literature review", International journal of energy research] and this can serve as a starting point for carrying out this addition. A research article on harmonic mitigation of PV systems has also been published in ["Predictive Adaptive Filter for Reducing Total Harmonics Distortion in PV Systems", Energies]. The author can choose to either discuss the harmonic mitigation effort in a new section or perform additional harmonic analysis.

Response: In response to the above problem, we have made the following changes:

（1）Carefully read "Advances in reduction of total harmonic distortion in solar photovoltaic systems: A literature review"; "Predictive Adaptive Filter for Reducing Total Harmonics Distortion in PV Systems" two articles on harmonic suppression of photovoltaic power generation systems. These two articles are of great help to this paper to research the mechanism of the impact of photovoltaic power generation systems on-grid energy efficiency and are cited in the title "Literature Review", respectively [16][17].
（2）Add literature [18][19][20] to the title " Literature Review" in the revised manuscript to research the use of different methods for photovoltaic grid-connected inverters to suppress harmonics.
（3）In the title "Analysis of the impact of grid-connected photovoltaic power generation systems on-grid energy efficiency", the impact of harmonics generated by grid-connected photovoltaic power generation systems on-grid energy efficiency is analyzed. Considering the following three aspects: 1) photovoltaic grid-connected inverters themselves have harmonic control function; 2) the contribution of the harmonic distortion rate of current to the energy efficiency of the power grid is the smallest; 3) the harmonic content generated by a single photovoltaic power station has little impact on the energy efficiency trend of the power grid, we can ignore the impact of photovoltaic power system harmonics on-grid energy efficiency.
（4）In the third-level title "Insufficiency of article research and prospects for future research", it is pointed out that this research ignores the insufficiency of research on the influence of harmonics generated by a single photovoltaic power station on the line loss of the power grid. However, it also pointed out the prospects of future research: in the actual power grid operation, the load is constantly changing, and the characteristics of the electricity load are different, which will affect the content of harmonic current in the power system, especially in the mode where the power system is connected to a large number of photovoltaic clusters. However, the superposition or offset of the effect of harmonics after different photovoltaic clusters are connected to the grid will have an impact on the energy consumption and reliability of power grid operation. Therefore, the degree of influence of photovoltaic power generation clusters on the dynamic energy efficiency change trend of the power grid needs to be further studied.

Response to the comments of Reviewer #2

Comment No.1:The main contribution of the article is not clearly highlighted.

Response: At the end of the title "Literature Review", the academic contribution and value of this research are introduced. Academic contributions and value are as follows:

（1）Taking the auxiliary service capability of the photovoltaic power generation system as the starting point, the mechanism of the reactive power service capability of the photovoltaic power generation system supporting the energy efficiency of the power grid operation is studied, and the effect of the reactive power supporting capability of the photovoltaic power generation system on the energy efficiency support of the power grid operation is planned to be verified. It provides decision-making reference for the planning and design, investment cost control, and energy efficiency management of the grid company before the photovoltaic power generation system is integrated into the power grid.
（2）Taking the supporting effect of the reactive power service of the photovoltaic power generation system on the grid operation energy efficiency as an example, it is intended to reveal the general law of the impact of renewable energy on the grid operation energy efficiency after grid connection, to prove that renewable energy has participated in the auxiliary services of the power system to the system, in order to prove the extent of renewable energy's impact on the energy efficiency of the system after participating in auxiliary services of the power system. It provides a theoretical basis for the construction of a market mechanism for auxiliary services containing renewable energy and transaction optimization decisions.
（3）The research on the supporting mechanism of a single photovoltaic power generation system to the grid energy efficiency and the dynamic energy efficiency analysis method of multi-temporal and spatial scales can be further applied to the dynamic energy efficiency analysis in the grid cluster mode such as photovoltaic cluster or wind and solar cluster. Through the coupling mechanism and division of different cluster modes research realize the power grid lean energy efficiency management, and provide ideas and methods for power grid energy conservation and loss reduction.

Comment No.2:The introduction is too long and as a reader, we may lose the main point of the article. Please revise it with a better flow that highlights the issue and the contribution of the research.

Response: The introduction has been revised as follows:

（1）Divide "Introduction" and "Literature Review" into two separate headings.
（2）The title of this paper is changed from "Research on Supporting Mechanism of Reactive Power Service of PV System to Grid Energy Efficiency Based on Multi-time and Space-time Operation" to "Research on Supporting Mechanism of Ancillary Service of PV System to Grid Energy Efficiency Based on Multi-time and Space-time Operation”. And add “The auxiliary service of photovoltaic power generation system is mainly to make full use of the remaining capacity of the inverter in the photovoltaic power generation system and the SVG device to provide reactive power for the system realize the balance of reactive power of the power system and support for the voltage stability of the system.” to the introduction
（3）In the introduction, the background and significance of “research on supporting mechanism of ancillary service of the PV system to grid energy efficiency” are added: the real-time balance of reactive power plays an important role in stabilizing market transaction electricity prices. Renewable energy represented by photovoltaic power generation is widely integrated into the power grid, researches on economic operation control, power quality, energy efficiency evaluation, optimal utilization of reactive power resources, energy conservation, and emission reduction, and their impact on grid stability after grid connection have achieved certain results. However, there is a lack of mechanism explanation for the degree of its impact on the energy efficiency of the power grid. Clarifying the relationship between the reactive power resources in the renewable energy power generation and the energy efficiency of the power grid operation can further clarify the room for improvement in the energy efficiency management of the power grid. Under the multi-temporal and spatial scales operation mode of the system, based on the supporting capability of auxiliary services of renewable energy, how market entities in the power system can make effective market trading strategies for changes in system energy efficiency to improve its income is an urgent problem for all entities to solve. Especially under the background that renewable energy has fully penetrated the power grid and the energy utilization structure has been transformed, it still needs theoretical basic research to mine the value of the reactive power resources of renewable energy based on the perspective of energy efficiency management to realize the optimal allocation of system resources and energy-saving and emission reduction of the power system.
（4）The literature review deletes the literature that is less relevant to the content of the article and has a long history. Retain [2][16][19][35][36] of the original manuscript, followed by [5][11][12][22][23] of the revised manuscript. The last three years of literature are added, and the literature review is reviewed. Research [1-7] studies the reactive power support capability of inverters and SVG devices in photovoltaic power system and their coordination and optimization. Research [8-12] focuses on comprehensive benefit and energy efficiency evaluation of photovoltaic power systems. Research [13-14] focuses on the efficient utilization, and carbon emission reduction of renewable energy power generation after renewable energy is connected to the power grid. Research [15-20] focuses on the power quality of the power system after renewable energy is connected to the grid. Sort out the literature and make a comparison table of differences between the research in this article and the existing main related literature, and intuitively see the research content, goals, main problems, and methods of the existing related literature, and highlight the contribution of this research.

Comment No.3:All figures’ quality are not acceptable, especially Fig 1. Please redraw this figure and enhance the fonts quality in others.

Response: The first picture in the revised manuscript has been redrawn as required, the resolution and pixels of the remaining pictures have been modified as required, and the font has been changed to the Times New Roman.

Comment No.4;Ref 2 is not cited in the text, and there is no any reference of last 3 years!!!

Response: The missing reference [2] has been added to the "Literature Review" of the revised manuscript, and it is the reference [5] in the revised manuscript, and the references in the past three years have been added, as shown in the following table:

| Time | 2021 | 2020 | 2019 |
| --- | --- | --- | --- |
| literature | [1][8][9][10][13][14] | [2][7][15][16][17] | [3][4][6] |

Comment No.5:Adding a table of abbreviation could clear many points to readers.

Response: A list of terminology abbreviations has been prepared and added to the supporting information as required, including terminology, electrical technology, related algorithms, and other vocabulary abbreviations.

Comment No.6:Table of comparison of conducted work and related works in literature will point out the improvement of the findings.

Response: The difference comparison table between the research work in this paper and the existing literature research work is given and placed in the literature review.

We tried our best to improve the manuscript and made some changes in the manuscript. These changes will not influence the content and framework of the paper. And here we did not list the changes but marked in red in “Revised Manuscript with Track Changes”.

We appreciate for Editors/Reviewers' warm work earnestly, and hope that the correction will meet with approval.

Once again, thank you very much for your comments and suggestions.

Yours sincerely,

Cui Yong
